# Polysialylation of Glioblastoma Cells Is Regulated by Autophagy Under Nutrient Deprivation

**DOI:** 10.3390/ijms26157625

**Published:** 2025-08-06

**Authors:** Sofia Scibetta, Giuseppe Pepe, Marco Iuliano, Alessia Iaiza, Elisabetta Palazzo, Marika Quadri, Thomas J. Boltje, Francesco Fazi, Vincenzo Petrozza, Sabrina Di Bartolomeo, Alba Di Pardo, Antonella Calogero, Giorgio Mangino, Vittorio Maglione, Paolo Rosa

**Affiliations:** 1 Department of Medical-Surgical Sciences and Biotechnologies, University of Rome “Sapienza”, Polo Pontino, C.so della Repubblica 79, 04100 Latina, Italy; sofia.scibetta@uniroma1.it (S.S.); marco.iuliano@uniroma1.it (M.I.); alessia.iaiza@uniroma1.it (A.I.); vincenzo.petrozza@uniroma1.it (V.P.); antonella.calogero@uniroma1.it (A.C.); giorgio.mangino@uniroma1.it (G.M.); 2 IRCCS Neuromed, Via Dell’Elettronica, 86077 Pozzilli, Italy; giuseppe.pepe@neuromed.it (G.P.); dipardoa@hotmail.com (A.D.P.); vittorio.maglione@neuromed.it (V.M.); 3 DermoLab, Department of Surgical, Medical, Dental and Morphological Sciences, University of Modena and Reggio Emilia, Via del Pozzo 71, 41125 Modena, Italy; elisabetta.palazzo@unimore.it (E.P.); marika.quadri@unimore.it (M.Q.); 4 Institute for Molecules and Materials, Radboud University, Heyendaalseweg 135, 6525 AJ Nijmegen, The Netherlands; thomas.boltje@ru.nl; 5 Department of Anatomical, Histological, Forensic & Orthopedic Sciences, Section of Histology & Medical Embryology, University of Rome “Sapienza”, Via A. Scarpa, 14-16, 00161 Rome, Italy; francesco.fazi@uniroma1.it; 6 ICOT, Istituto Chirurgico Ortopedico Traumatologico, Via F. Faggiana 1668, 04100 Latina, Italy; 7 Department of Biosciences and Territory, University of Molise, Contrada Fonte Lapone, 86090 Pesche, Italy; sabrina.dibartolomeo@unimol.it

**Keywords:** glioblastoma, polysialic acid, nutrient deprivation, tumor microenvironment, autophagy

## Abstract

Glioblastoma (GBM) is a highly aggressive brain tumor marked by invasive growth and therapy resistance. Tumor cells adapt to hostile conditions, such as hypoxia and nutrient deprivation, by activating survival mechanisms including autophagy and metabolic reprogramming. Among GBM-associated changes, hypersialylation, particularly, the aberrant expression of polysialic acid (PSA), has been linked to increased plasticity, motility, and immune evasion. PSA, a long α2,8-linked sialic acid polymer typically attached to the NCAM, is abundant in the embryonic brain and re-expressed in cancers, correlating with poor prognosis. Here, we investigated how PSA expression was regulated in GBM cells under nutrient-limiting conditions. Serum starvation induced a marked increase in PSA-NCAM, driven by upregulation of the polysialyltransferase ST8SiaIV and an autophagy-dependent recycling of sialic acids from degraded glycoproteins. Inhibition of autophagy or sialidases impaired PSA induction, and PSA regulation appeared dependent on p53 function. Immunohistochemical analysis of GBM tissues revealed co-localization of PSA and LC3, particularly around necrotic regions. In conclusion, we identified a novel mechanism by which GBM cells sustain PSA-NCAM expression via autophagy-mediated sialic acid recycling under nutrient stress. This pathway may enhance cell migration, immune escape, and stem-like properties, offering a potential therapeutic target in GBM.

## 1. Introduction

Glioblastoma IDH-wildtype (GBM, WHO grade 4) is the most aggressive form of glioma characterized by the highest incidence and a median survival of about 18 months for patients who receive complete surgical removal, radio- and chemotherapy [1].

The standard treatment for all GBM patients is temozolomide (TMZ), a methylating agent derived from dacarbazine. TMZ acts by slowing cancer cell growth interfering with cell cycle and triggering apoptosis [2]. Nevertheless, GBM invariably recurs, due to its propensity to infiltrate the surrounding parenchyma [3].

The tumor microenvironment (TME) of GBM represents a highly heterogeneous and dynamic system composed of endothelial cells, neurons, astrocytes, oligodendrocytes, and resident and circulating immune cells [4]. TME is strongly influenced by alterations in cellular composition and cellular metabolic products as well as other chemical factors such as hypoxia, consisting in low oxygen levels generated due to the rapid proliferation of tumor cells.

Hypoxia plays a central role in enhancing drug resistance in GBM cells by promoting the acquisition of a stem phenotype [5] and affecting their growth, invasiveness, and migration [6]. Neoplastic cells preferentially use aerobic glycolysis (Warburg effect), by converting glucose to lactic acid, even in the presence of O_2_. This results in abnormal proliferation of cancer cells that facilitates malignant progression [7]. Concomitantly, nutrient deprivation within the TME activates adaptive responses that mitigate apoptosis and support tumor cell survival [8], while also modulating invasive behavior [4].

One of the main mechanisms induced by low nutrient conditions is autophagy, a conserved cellular process of organelle recycling that is crucial for maintaining homeostasis [9]. In GBM cells, autophagy induces migration and chemoresistance by sustaining cellular energetics [9].

The aggressiveness of cancer cells is strongly influenced by the TME, which affects cellular metabolism and shapes the dense layer of multifunctional glycans on the cell surface, known as the glycocalyx.

Described as a subclass of the glycome, the sialome has been likened to a dense forest covering the cell membrane, playing crucial roles in cell–cell interactions [10].

A unique class of glycans is represented by sialic acids, monosaccharides characterized by different types of linkages (α2,3-, α2,6-, and α2,8-) which strongly influence the structure and function of proteins and lipids [11]. Aberrant sialylation plays a key role in the interaction between glioma cells and the surrounding microenvironment, promoting tumor development and progression [12].

The Neural Cell Adhesion Molecule (NCAM) mediates homophilic cell adhesion and regulates migration; its function is critically governed by polysialylation [13,14].

Polysialic acid (PSA) is a carbohydrate consisting of linear sialic acid chains linked by α2,8-glycosidic bonds [15]. Many cancers are characterized by cells displaying hypersialylation, which can occur not only through upregulation of sialyltransferase activity but also due to dysregulation of neuraminidases, enzymes that remove sialic acid residues from cell surface glycans. PSA has been recognized as an oncodevelopmental antigen being present at high levels during embryogenesis and early life stages, downregulated in the adult, and re-expressed in tumors [16,17]. High expression of PSA-NCAM is associated with high-grade, low-differentiated tumors and the ability to spread aggressively [18].

Although the critical role of tumor cell–microenvironment interactions is well established, the contribution of PSA to GBM pathogenesis and TME adaptation remains poorly understood.

Here, we examined PSA metabolism in GBM cells under nutrient deprivation, a defining feature of the TME that drives tumor aggressiveness.

## 2. Results

### 2.1. STs, NEUs, and CMAS Expression in GBM Cells

Hypersialylation is a hallmark of aggressive cancers [10,19]; however, its relevance in glioblastoma remains underexplored. Therefore, we assessed mRNA levels of key sialylation enzymes in two established GBM cell lines (U87-MG, U251), multiple patient-derived glioma cultures (low grade: GL18-1, GL18-3; high grade: GL18-2, 4, 5, 7, 15), and a brain metastasis.

**Figure 1:**
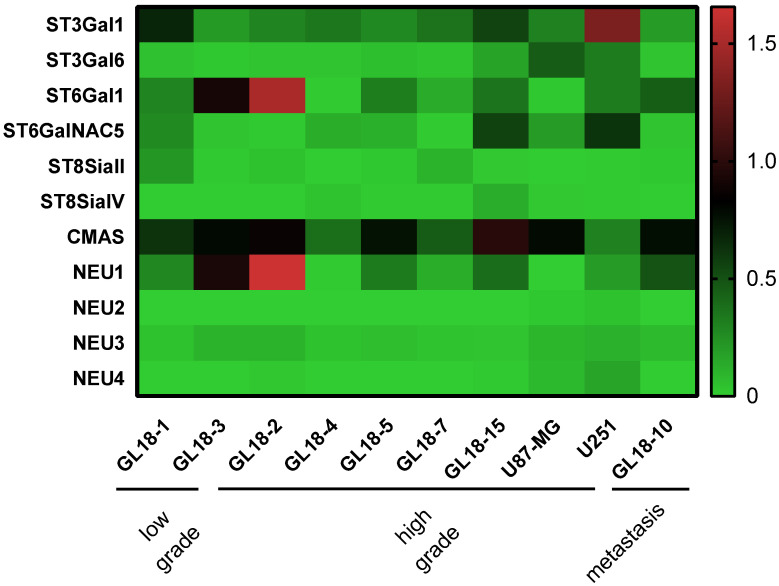
Expression profile of sialyltransferases, polysialyltransferases, cytidine monophosphate N-acetylneuraminic acid synthetase, and neuraminidases in glioblastoma cells. Real-time analysis showing mRNA expression of sialyltransferases (ST3Gal1, ST3Gal6, ST6Gal1, ST6GalNAC5), polysialyltransferases (ST8SiaII, ST8SiaIV), cytidine monophosphate N-acetylneuraminic acid synthetase (CMAS), and neuraminidases (NEU1-4) in human low-grade (GL18-1 and GL18-3) and high-grade (GL18-2, GL18-4, GL18-5, GL18-7, and GL18-15) glioma patient-derived primary cultures compared to U87-MG and U251 GBM cell lines and a kidney metastasis to the brain. Expression values were normalized by assigning the lowest value across all samples as the baseline reference.

Sialyltransferase transcripts (ST3Gal1, ST3Gal6, ST6Gal1, ST6GalNAC5) exhibited marked variability; notably, ST3Gal1 and ST6Gal1 were the most expressed among GBM cultures (Figure 1). CMAS, which converts Neu5Ac to CMP–sialic acid, was uniformly elevated across all GBM models. Among the neuraminidases (NEU1–4), NEU1 was abundantly expressed in almost all primary cultures, U251, and the metastasis but low in U87-MG and GL18-4 cells.

### 2.2. Serum Deprivation Induces PSA Levels in GBM Cells

**Figure 2:**
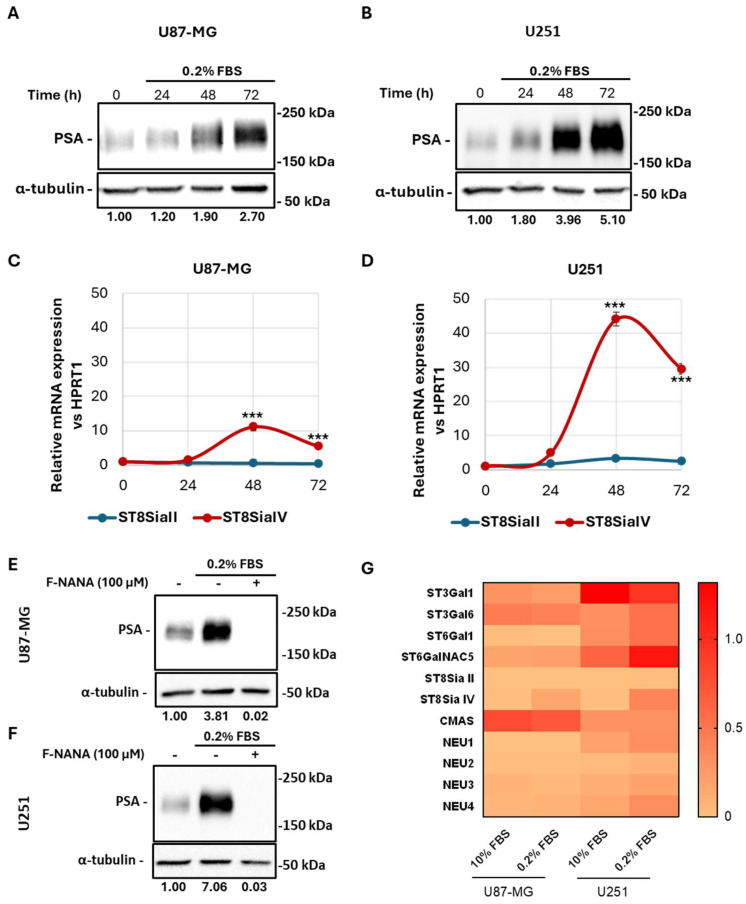
Effects of serum starvation on the expression of PSA, PolySTs, STs, and NEUs in U87-MG and U251 cells. Western blot analysis showing the time course (0–72 h) of PSA expression in U87-MG (**A**) and U251 (**B**) cells exposed to serum deprivation. (**C**) Real-time PCR analysis showing the time course (0–72 h) of mRNA expression of polysialyltransferases ST8SiaII and ST8SiaIV in U87-MG (**C**) and U251 (**D**) cells exposed to serum deprivation. Western blot analysis of PSA in U87-MG (**E**) and U251 (**F**) cells exposed to serum deprivation, in the presence or not of 100 μM F-NANA. (**G**) Real-time PCR analysis showing mRNA expression of sialyltransferases (ST3Gal1, ST3Gal6, ST6Gal1, ST6GalNAC5), polysialyltransferases (ST8SiaII, ST8SiaIV), cytidine monophosphate N-acetylneuraminic acid synthetase (CMAS), and neuraminidases (NEU1-4) in U87-MG and U251 cells exposed (0.2% FBS) or not (10% FBS) to serum deprivation for 48 h. Expression values were normalized by assigning the lowest value across all samples as the baseline reference. Results are presented as the mean ± SD of three independent experiments. ***: *p* < 0.001.

To mimic nutrient stress in the tumor microenvironment [4], U87-MG and U251 cells were cultured in 0.2% versus 10% FBS. Immunoblotting revealed a 2.7-fold and 5.1-fold increase in NCAM polysialylation at 72 h in U87-MG and U251 cells, respectively (Figure 2A,B). This PSA induction followed robust upregulation of ST8SiaIV transcripts (about 11-fold in U87-MG cells, about 40-fold in U251 cells at 48 h; Figure 2C,D). Treatment with 100 μM F-NANA (a cell-permeable sialic acid analog, able to competitively inhibit polysialyltransferases) abolished starvation-induced PSA in both lines (Figure 2E,F). Under 48 h starvation, U251 cells further increased ST3Gal6, ST6Gal1, and ST6GalNAC5, whereas U87-MG cells showed modest reductions, except for ST6GalNAC5; both cell lines downregulated ST3Gal1 (Figure 2G). CMAS expression remained stable, and only U251 cells altered NEU isoforms.

### 2.3. Serum Starvation Preferentially Induces Cell Surface NCAM Polysialylation While Reducing Other Proteins’ Sialylation in GBM Cells

**Figure 3:**
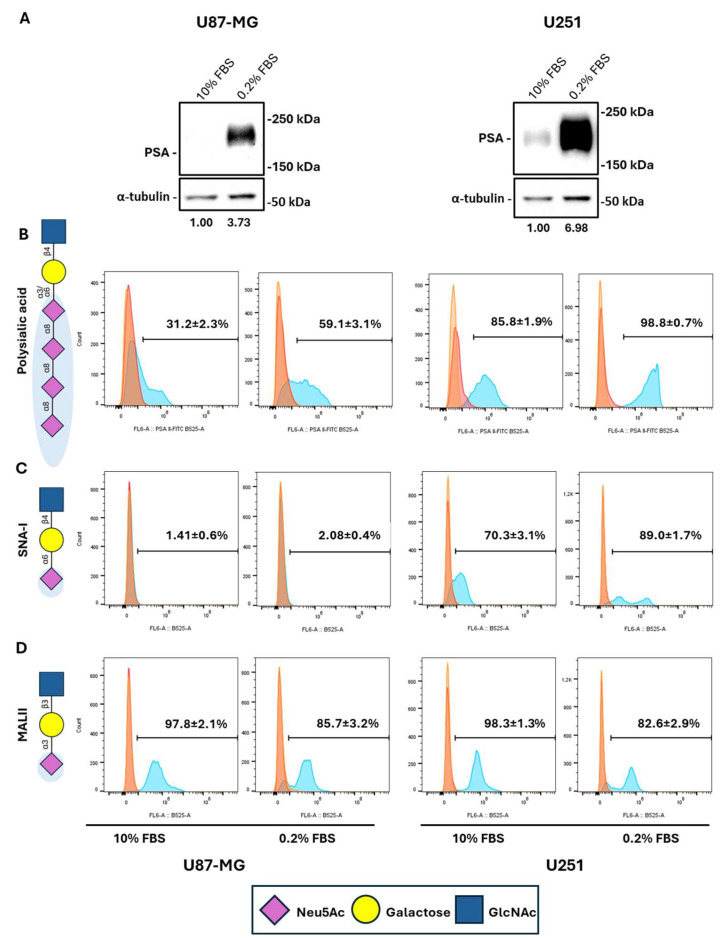
Effects of serum starvation on the extracellular expression of α2-3-, α2-6-linked sialic acids and PSA in U87-MG and U251 cells. (**A**) Western blot analysis showing PSA expression in U87-MG and U251 cells exposed to serum starvation for 72 h. (**B**) Cytofluorimetric analysis showing the extracellular expression of polysialic acid in U87-MG and U251 cells exposed or not for 72 h to 0.2% FBS. Cytofluorimetric analysis showing the extracellular expression of α2-6-linked sialic acids detected by SNA-I lectin binding (**C**) and α2-3-linked sialic acids detected by MALII lectin binding (**D**) in U87-MG and U251 cells exposed or not for 72 h to serum starvation. Results are presented as the mean ± SD of three independent experiments. Light orange histogram: autofluorescence; orange histogram: secondary antibody; light blue histogram: polysialic acid, SNA-I and MALII stainings. Legend represents the monomers that constitute the sialic acid chains (galactose, Neu5Ac: N-acetylneuraminic acid; GlcNAc: N-acetylglucosamine). Created with BioRender.com.

Beyond PSA, the sialome includes several sialic acids associated with various glycoproteins or glycolipids and plays a critical role in the intricate architecture of cell membranes. Aberrant sialylation has been shown to impact malignant cells’ behavior [20]. As shown in Figure 3A, total NCAM polysialylation was increased in both U87-MG and U251 cells after 72 h of serum deprivation. Flow cytometry demonstrated that serum starvation increased PSA-positive cells (31.2% ± 2.3 under 10% FBS vs. 59.1% ± 3.1 under 0.2% FBS for U87-MG cells, *p* < 0.001; 85.8% ± 1.9 under 10% FBS vs. 98.8% ± 0.7 under 0.2% FBS for U251 cells, *p* < 0.001) (Figure 3B). Also, we evaluated the abundance of other extracellular α2,6- and α2,3-linked sialic acids by SNA-I and MALII lectins staining. SNA-I lectin staining showed negligible α2,6-linked sialic acids in U87-MG cells and that serum deprivation was not able to significantly upregulate cell positivity. Conversely, a larger subpopulation of U251 cells displayed α2,6-linked sialic acids on their plasma membrane, which appreciably increased in response to serum deprivation (70.3% ± 3.1 under 10% FBS vs. 89.0% ± 1.7 under 0.2% FBS, *p* < 0.001) (Figure 3C). MAL-II staining revealed α2,3-linked sialic acids were abundantly expressed on the cell surface of both U87-MG (97.8% ± 2.1) and U251 (98.3% ± 1.3) cells and that serum deprivation significantly reduced both cell lines positivity (85.7% ± 3.2 for U87-MG cells, *p* < 0.01; 82.6% ± 2.9 for U251 cells, *p* < 0.001) (Figure 3D).

### 2.4. Nutrient Deprivation-Induced PSA Levels Are Regulated by Autophagy in GBM Cells

**Figure 4:**
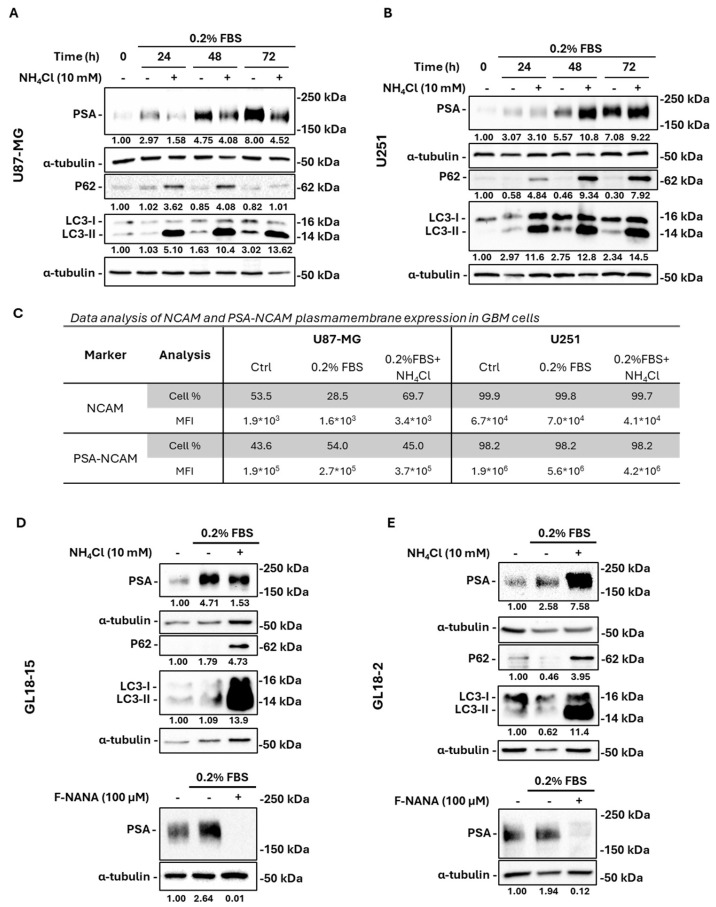
Effects of serum deprivation-induced autophagy on PSA expression in GBM cell lines and primary cultures. Western blot analysis showing the expression of PSA and the autophagic related proteins LC3-I/II and p62 in U87-MG (**A**) and U251 (**B**) cells exposed to serum deprivation for 24, 48, and 72 h in the presence or not of 10 mM NH_4_Cl. (**C**) Cytofluorimetric analysis of the extracellular expression of the NCAM and PSA-NCAM in U87-MG and U251 cells exposed for 72 h to serum deprivation in the presence or not of 10 mM NH_4_Cl. The table shows the positivity to both markers (expressed as a percentage) and the Mean Fluorescence Intensity (MFI). Western blot analysis showing the expression of PSA and the autophagy-related proteins LC3-I/II and p62 in GL18-15 (**D**) and GL18-2 (**E**) GBM primary cultures exposed to serum deprivation for 72 h in the presence or not of 10 mM NH_4_Cl. The reported LC3 densitometric analysis referred to the LC3-II isoform. Bottom panels show representative images of PSA expression in both GBM primary cultures exposed for 72 h to serum deprivation, in the presence or not of 100 μM F-NANA. Results are expressed as the mean ± SD of three independent experiments.

Nutrient deficiency has a profound effect on GBM tumor biology by interfering with signaling pathways that govern cell proliferation and drug resistance mechanisms, with autophagy being a key factor [21,22]. Under starvation, PSA accumulation paralleled LC3-II induction and p62 degradation over 0–72 h (Figure 4A,B). Inhibition of autophagy with 10 mM NH_4_Cl, shown by LC3-II and p62 accumulation, exerted divergent effects: PSA decreased in p53^wt^ U87-MG cells, while it increased in p53^mut^ U251 cells (Figure 4A,B). Cytofluorimetry confirmed that nutrient deprivation in U87-MG cells increased the extracellular expression of PSA (43.6% ± 3.2 ctrl vs. 54.0% ± 2.7 under 0.2% FBS, *p* < 0.01). This increase was prevented by autophagy inhibition with NH_4_Cl (dropping to 45.0% ± 1.9 under 0.2% FBS + NH_4_Cl, *p* < 0.01). PSA changes were inversely correlated with NCAM cell surface expression. Under nutrient deprivation, NCAM positivity decreased significantly (53.5% ± 4.3 ctrl vs. 28.5% ± 2.0 under 0.2% FBS, *p* < 0.001), but autophagy inhibition restored and even enhanced NCAM levels (up to 69.7% ± 4.9 under 0.2% FBS + NH_4_Cl, *p* < 0.001). In contrast, U251 cells exhibited near-constitutive surface expression of both the NCAM and PSA. Under serum starvation, PSA Mean Fluorescence Intensity (MFI) significantly increased (1.9 × 10^6^ ± 1.1 × 10^5^ ctrl vs. 5.6 × 10^6^ ± 2.9 × 10^5^ under 0.2% FBS, *p* < 0.001), and this was partially reduced by NH_4_Cl (to 4.2 × 10^6^ ± 2.0 × 10^5^, *p* < 0.01). However, this modulation was not associated with increased NCAM surface levels, which actually decreased (7.0 × 10^4^ ± 2.3 × 10^3^ under 0.2% FBS vs. 4.1 × 10^4^ ± 1.6 × 10^3^ under 0.2% FBS + NH_4_Cl, *p* < 0.01) (Figure 4C). Parallel experiments in primary cultures (GL18-15 p53^wt^ vs. GL18-2 p53^mut^) recapitulated these p53-dependent effects (Figure 4D,E). F-NANA maintained its ability to abolish PSA induction under nutrient deprivation in both GBM primary cultures.

### 2.5. Neuraminidases Activity Is Fundamental for PSA Turnover in Serum-Deprived GBM Cells

**Figure 5:**
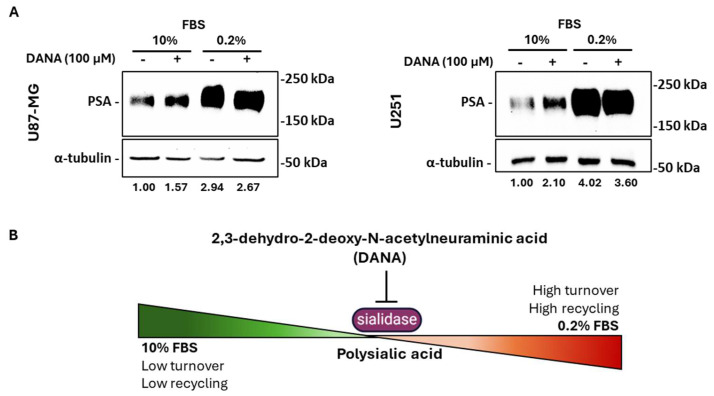
Effects of sialidases’ inhibition on PSA expression in nutrient-deprived GBM cells. (**A**) Western blot analysis showing expression of PSA in U87-MG and U251 cells exposed or not to serum deprivation for 72 h in the presence or not of 100 μM DANA. (**B**) Scheme showing the role of sialidases in PSA dynamics. Specifically, in normal conditions, PSA expression is maintained by the balanced activity of sialyltransferases and sialidases. In serum starvation conditions, the high recycling rate of other sialic acids due to augmented autophagy relies on sialidases’ activity for PSA constant turnover.

PSA turnover reflects a balance between sialyltransferases and sialidases [19]. In order to evaluate the involvement of neuraminidases in the modulation of PSA levels under nutrient-deprived conditions, we treated GBM cells with DANA, a sialidase inhibitor. Under normal condition, treatment with 100 μM DANA increased PSA, especially in U251 cells, whereas, under serum starvation, DANA attenuated PSA induction, confirming that neuraminidase-mediated recycling is essential for sustaining PSA levels when nutrient availability is low (Figure 5A,B).

### 2.6. PSA Expression Is Related to Autophagy Activation in GBM Tissues

**Figure 6:**
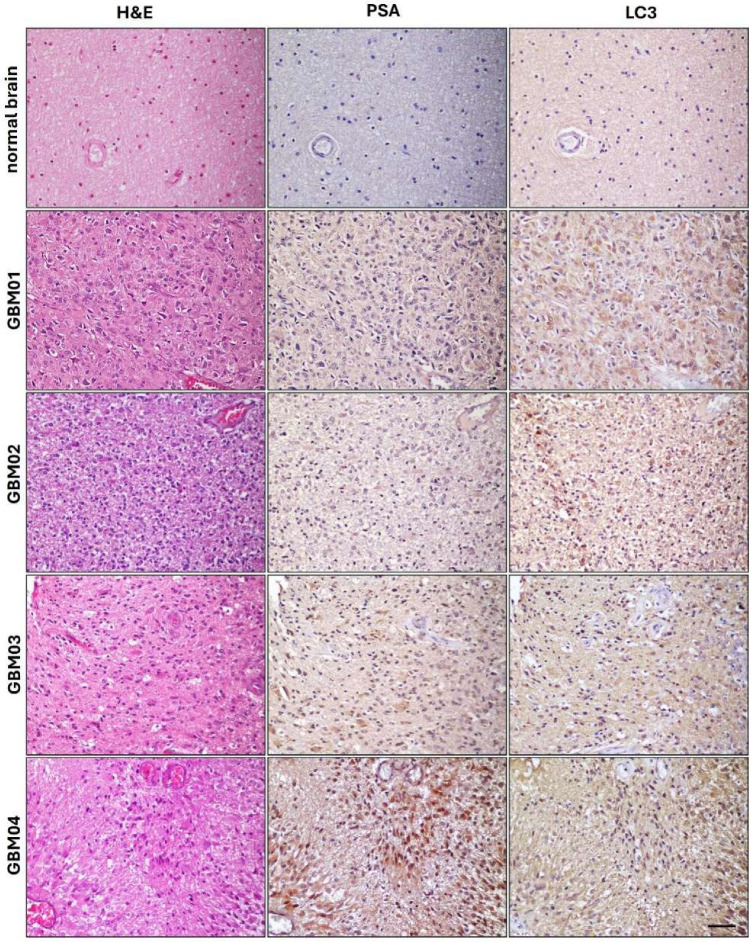
PSA and LC3 expression in GBM tissues. Immunohistochemical analysis showing PSA and LC3 expression and localization in formalin-fixed paraffin-embedded serial sections from GBM patients (GBM01–04) compared to normal brain tissue. Brown staining indicates positive immunoreactivity. Hematoxylin/eosin (H&E) stained sections were included for histological evaluation. Magnification 20×. Scale bar: 50 μm.

The autophagic process is upregulated in tumors favoring the recycling of many components when cells are exposed to extreme environments [23]. Figure 6 illustrates the immunohistochemical analysis of PSA and the autophagy marker LC3 in GBM tissues. PSA was broadly expressed, with variable intensity among patients, predominantly localized in the cytoplasm. Notably, PSA was strongly upregulated around necrotic areas, especially in the GBM04 sample. LC3 expression was observed in the same regions on serial sections, suggesting co-localization. In contrast, normal brain tissues showed minimal PSA and faint LC3 staining. Hematoxylin/eosin staining confirmed typical GBM features, including high cellularity, pleomorphism, microvascular proliferation, and pseudo-palisading cells around necrotic areas. Overall, data suggested a correlation between PSA expression and autophagy activation in GBM.

## 3. Discussion

The main findings reported in this study highlight that (i) nutrient deprivation induces NCAM polysialylation in GBM cells; (ii) serum starved GBM cells upregulate cell surface PSA while downregulating other sialic acids; (iii) sialic acid analogs are effective in avoiding serum starvation-induced PSA synthesis in GBM cells; and (iv) autophagy plays a pivotal role in regulating PSA turnover by recycling other sialic acids in GBM cells under nutrient deprivation conditions.

Tumor glycobiology has always attracted interests because modifications of the cell surface greatly influence cell function [24]. Cancer cell membranes display altered composition in lipids and proteins, which are aberrantly glycosylated [25]. Among the many glycans, sialic acids’ overexpression has been demonstrated to promote tumor cells aggressiveness by the acquisition of a more invasive phenotype and the ability to escape immune surveillance [26]. These features occur in specific areas of the tumor, taking advantage of extreme environments.

Sialic acids play well-characterized functions in tumors such as breast cancer [27], melanoma [28], and lung cancer [29], whereas their role in GBM has been poorly investigated. Our group already demonstrated that the expression of a unique type of sialic acid, PSA, played a role in the hypoxia-induced aggressiveness of GBM cells by sustaining their migration and undifferentiated state [30]. Here, we reported a role for PSA in GBM cells exposed to another condition mimicking the TME, nutrient deprivation, by focusing on the mechanisms underlying its turnover and synthesis dynamics.

Our observations highlighted that all the enzymes involved in protein sialylation in low- and high-grade glioma were variably expressed among the tested cultures, showing a prevalence of α2,3- and α2,6- sialyltransferases expression. This result underlies the importance of protein sialylation in cancer and is in line with other studies analyzing sialic acids in other tumors [31,32,33]. Sialyltransferases’ activity has also been associated with the invasive and migratory capacity of cancer cells [34,35]. Interestingly, the expression of the brain-specific ST6GalNAC5 enzyme by breast cancer cells was found to mediate brain metastases by enhancing blood–brain barrier crossing [36]. Among the analyzed enzymes, CMAS was abundantly expressed in all patients, thus underlying its central role in sustaining sialylation by constantly providing activated sialic acid monomers to the different sialyltransferases [37]. Then, the evaluation of neuraminidases’ expression revealed high levels of the lysosomal NEU1, which suggests a potential dependence of GBM cells on its sialic acids’ breakdown function for de novo sialylation [38,39]. Surprisingly, the levels of polysialyltransferases were very low compared to the other sialyltransferases. This could be explained by the large number of sialylated proteins compared to the few polysialylated ones [40,41]. However, in this study, we observed an increase in the ST8SiaIV transcripts only when GBM cells were exposed to a nutrient-deprived microenvironment, as shown by in vitro experiments performed with U87-MG and U251 cell lines. By contrast, the analyzed GBM cultures barely expressed ST8SiaII, which plays a pivotal role during brain development, whereas ST8SiaIV mainly regulates PSA synthesis in the adult brain [42]. Moreover, polySTs’ activity is associated with the length of PSA chains, which may be required for different functions or conditions [43,44]. The reasons for the observed variability among the presented GBM cultures cannot exclude the different genetic background, mutational status, and/or tumor differentiation degree.

The increase in the ST8SiaIV transcript levels that we described under serum deprivation in GBM cells was accompanied by the increase in PSA-NCAM protein expression, representing the main finding presented in this study. Two main questions arise from our observations: why should a cancer cell use energy to synthesize PSA in energy-saving situations? How can a cancer cell reach this goal? Given the well-characterized role of PSA in cell migration [45,46,47], the explanation for this result could be the need of GBM cells to escape from extreme microenvironments, as we have already demonstrated under hypoxic conditions [30]. The answer to the second question should consider the specific cellular mechanism activated when energy levels are low, allowing synthesis solely through recycling: autophagy. A clear indication supporting our hypothesis comes from our results when we inhibited polySTs’ activity by F-NANA administration under nutrient deprivation. The mode of action of this molecule is to competitively block de novo protein and lipid sialylation. Studies by Büll and colleagues demonstrated the efficacy of this fluorinated sialic acid mimetic (also referred to as Ac53FaxNeu5Ac) in suppressing melanoma growth by enhancing T-cell-mediated tumor immunity [48] and inhibiting the melanoma’s metastatic spread to lungs [49]. In our previous work, we also demonstrated the efficacy of its intranasal administration in reducing PSA levels in the mouse brain [30]. Moreover, in BRCA1-mutated breast cancers, this sialyltransferase inhibitor was reported to neutralize acidic tumor-permissive microenvironment and sensitize cancer cells to immune checkpoint blockade by activating CD8^+^ T cells and inhibiting tumor growth and metastasis [50]. Our results show a complete abolishment of PSA levels that were induced under serum starvation, thus indicating a constant turnover for PSA-NCAM post-translational modification. Furthermore, where do GBM cells under nutrient deprivation take sialic acids from? Our cytofluorimetric analyses demonstrated that the increase in the total levels of PSA expression reflected its levels on the cell surface. Moreover, the lectin binding assays that we performed suggested α2,3-linked sialic acids as the source for PSA synthesis in nutrient-deprived GBM cells. Surprisingly, U87-MG cells seemed to be negative for α2,6-linked sialic acids, while U251 cells upregulated this type of extracellular modification similarly to PSA in low-serum conditions. To note, U251 cells were also able to upregulate all the machinery for sialylation and polysialylation at higher levels compared to U87-MG cells and this could be due to its p53 mutational status. Indeed, the p53^R273H^ mutation, carried by U251 cells, was also described to promote non-small cell lung cancer (NSCLC) cell migration by upregulating NEU1 [51].

Our experiments inhibiting the autophagic process provided a proof of concept for the role of autophagy in PSA synthesis under nutrient deprivation in GBM cells. Autophagy has been demonstrated to drive GBM drug resistance [21] and to regulate cell migration and invasion [52]. Hence, targeting this process is considered a promising therapeutical intervention. Since the involvement of autophagy in the dynamics of PSA production has been poorly investigated, we chose to block this process with ammonium chloride (NH_4_Cl), which elevates lysosomal pH preventing proteases activity [53]. We demonstrated that the treatment of U87-MG and U251 cell lines with NH_4_Cl under serum starvation had opposite effects on total cell polysialylation. This result was also confirmed by two different GBM patient-derived primary cultures (GL18-2 and GL18-15); the different effects observed on PSA expression following autophagy inhibition under serum deprivation seemed to be dependent on the p53 mutational status. Indeed, p53^wt^ cells (U87-MG and GL18-15) displayed a significant decrease in PSA levels under nutrient-deprived conditions upon autophagy blockage, while its accumulation as observed in p53^mut^ GBM cells (U251 and GL18-2). These observations open new insights into a p53-dependent mode of action exploited by GBM cells, which need to recycle sialic acid under extreme conditions to guarantee NCAM post-translational modification. The dependance of autophagy on p53 function was already described in cancer cells [54,55,56]. The differential response to autophagy inhibition observed between wild-type and mutant p53-expressing cells likely reflects the multifaceted and context-dependent roles of p53 in regulating autophagy, metabolism, and cell fate. Wild-type p53 can exert dual effects on autophagy depending on its subcellular localization and interactions with key signaling molecules. Nuclear p53 generally promotes autophagy through transcriptional activation of autophagy-related genes such as DRAM1, while cytoplasmic p53 has been shown to suppress autophagy through non-transcriptional mechanisms, involving direct interactions with the autophagy machinery and regulation of metabolic sensors like AMPK and mTOR [57,58,59]. In contrast, mutant p53 proteins often lose these regulatory capabilities or acquire gain-of-function activities that interfere with cellular stress responses and promote tumor progression. This altered signaling landscape may render mutant p53-expressing cells less responsive or differently sensitive to autophagy inhibition. Although our study was not primarily designed to dissect the downstream signaling pathways of p53 in this context, these findings underscore the importance of the p53 status in modulating autophagic responses and highlight a potential avenue for therapeutic stratification. To better understand our results, we also analyzed the extracellular levels of PSA in these cells and found that NH_4_Cl was always effective in reducing cell surface NCAM polysialylation under low-nutrient conditions (see also Supplementary Figure S1). In U87-MG cells, where p53 function was not compromised, the total decrease in PSA expression was associated with a cell surface decrease in PSA and a concomitant increase in NCAM expression when nutrient-deprived cells were exposed to NH_4_Cl. In that situation, extracellular PSA-NCAM could be subjected to the membrane neuraminidase NEU3 proteolytic activity during its internalization into recycling vesicles to guarantee its turnover. On the contrary, in p53^mut^ U251 cells, the described accumulation of PSA-NCAM when an active autophagic process was blocked may be caused by the internalization of the entire polysialylated form. The differential PSA response to autophagy inhibition observed in the two GBM models suggests the involvement of multiple, potentially mutation-specific mechanisms regulating PSA processing, trafficking, or synthesis. These findings underscore the complexity of PSA regulation in the context of p53 status and highlight the need for further studies to elucidate the molecular pathways involved. However, in both scenarios, we cannot rule out the involvement of various types of vesicles (e.g., early endosomes, late endosomes/multivesicular bodies, autophagosomes), which ultimately converge at lysosomes to facilitate sialic acid recycling under nutrient-deprived conditions [60]. Lysosomes are essential for the degradation and recycling of cellular components during the process of autophagy [61]. In these organelles, neuraminidases are also localized, specifically NEU1, whose correct function and activity is intricately linked to the lysosomal environment [62]. Sialin, a transporter of sialic acids, is also present in lysosomes, and its stability is regulated by autophagy [63]. Another evidence of the involvement of autophagy in sialylation was described in CHO cells, where the inhibition of this process caused a significant impairment of sialylation, which could be restored by nucleotide sugar precursors’ administration particularly under mild stress conditions [64]. Moreover, serum starvation represents the typical condition enabling cellular autophagy [65], and it has been demonstrated that nutrient-deprived breast cancer cells rely on sialic acids to maintain good levels of cell surface glycosylation [66].

In normal conditions, the function of sialidases is to proteolytically remove sialic acid residues on proteins and lipids, thus antagonizing the role of sialyltransferases to control cell sialylation levels [67]. Kuliesiute and colleagues described a significant turnover of sialic acids in GBM cells and that interfering with sialidases and sialyltransferases affected tumor growth and cell connectivity [68]. Accordingly, when we treated GBM cells with 100 μM DANA [68,69,70], a sialidase inhibitor, we measured an increase in PSA levels. However, our results clearly demonstrated that when we blocked sialidases’ activity in low-nutrient conditions, we observed a reduction in PSA levels, suggesting again that nutrient deprivation pushes the cell into a high recycling mode, which implies a high turnover for PSA guaranteed by neuraminidases’ activity.

The association of PSA expression and autophagic activation was also observed directly on GBM tissues by immunohistochemical analyses, evidencing the physiopathological relevance of our study. We are aware that LC3 expression may not be the ideal marker for autophagy activation because the antibody does not discriminate among the different I and II isoforms. However, it has already been demonstrated that LC3 expression correlates with GBM tumor grade, with grade 4 expressing it the most [71]. We also noticed that PSA was markedly expressed around necrotic areas by pseudo-palisading cells, suggesting the influence of extreme microenvironments in inducing PSA expression.

**Figure 7:**
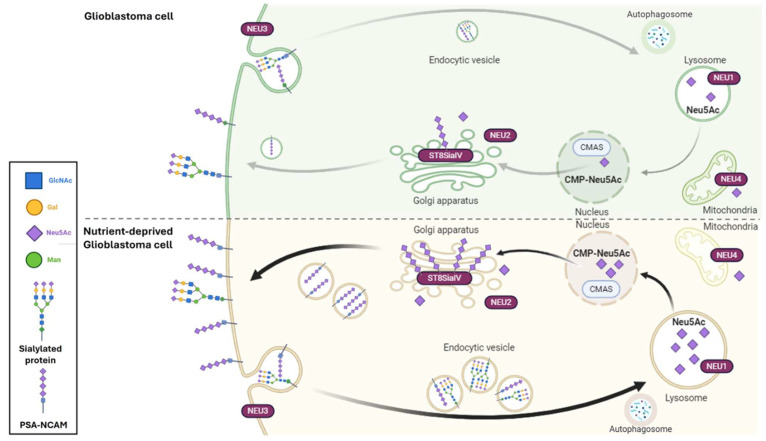
Proposed mechanism for nutrient deprivation-induced NCAM polysialylation in GBM cells. The scheme evidences the dynamics of PSA turnover in GBM cells, which is constantly synthesized from the recycling of other sialic acids glycoconjugated to membrane proteins and lipids. When nutrients are normally available, PSA levels are maintained by a slow rate of sialic acids’ recycling and balanced activity of neuraminidases and polysialyltransferases. In conditions of low nutrients, the activation of the autophagic process and the increased activity of neuraminidases bring to lysosomes high quantities of sialic acid monomers, which are activated in the nucleus by the CMAS enzyme and, once in the Golgi apparatus, can be elongated in long chains of polysialic acid and attached to NCAM proteins by augmented polysialyltransferases for cell surface mobilization. Created with BioRender.com.

The proposed mechanism for NCAM polysialylation dynamics in GBM cells under serum starvation is summarized in Figure 7. When GBM cells experience low-nutrient conditions, the normal extracellular composition in sialylated proteins and lipids becomes a source for the elongation of long chains of PSA. The identified mechanism is the autophagic recycling of membrane glycoconjugates that need to reach the lysosomes by vesicular trafficking. Here, NEU1 activity sustains the high turnover of PSA by making sialic acid monomers available to be activated in the nucleus by the CMAS enzyme. At this point, activated sialic acid residues can be presented to ST8SiaIV in the Golgi apparatus for polymerization and mobilization of the de novo polysialylated NCAM to the plasma membrane.

In conclusion, this study provides evidence of novel potential mechanisms sustaining GBM cell polysialylation under low-nutrient conditions. Upregulating cell surface polysialylation could give GBM cells the advantage to maintain stemness, escape extreme microenvironments, and avoid immune surveillance. Every part of the described machinery involved in this “polysialylation mission” could represent a potential target for therapeutical interventions to limit the aggressive behavior of a lethal tumor such as GBM.

## 4. Materials and Methods

### 4.1. Cell Cultures and Reagents

U87-MG and U251 human glioblastoma multiform cell lines were purchased from CLS (Cell Lines Service GmbH, Eppelheim, Germany). GBM cell lines were grown in Dulbecco’s Modified Eagle Medium (DMEM) supplemented with 10% heat-inactivated Fetal Bovine Serum (FBS, Sigma-Aldrich, St. Louis, MO, USA), 100 IU/mL penicillin G, 100 µg/mL streptomycin, 1% L-glutamine, 1% nonessential amino acids, and 1 mM sodium pyruvate at 37 °C in 5% CO_2_-humidified atmosphere. The cells were sub-cultured only when confluent, and the medium was replaced twice a week. GBM primary cultures’ establishment and molecular characterization have already been described by our group [30,72]. Primary cultures were grown in Dulbecco’s Modified Eagle Medium (DMEM)/Ham’s F12 medium (1:1) supplemented with 10% heat-inactivated Fetal Bovine Serum (FBS, Sigma-Aldrich, St. Louis, MO, USA), 100 IU/mL penicillin G, 100 µg/mL streptomycin, 1% L-glutamine, 1% nonessential amino acids, and 1 mM sodium pyruvate at 37 °C in 5% CO_2_-humidified atmosphere. Culture medium was half-changed every three/four days, and the cells were sub-cultured only when total confluence was reached. The sialyltransferase inhibitor F-NANA (3Fax-Peracetyl Neu5Ac, 566224, Sigma-Aldrich) was dissolved in dimethyl sulfoxide (DMSO, Sigma-Aldrich) at a final concentration of 100 mM. The neuraminidase inhibitor DANA (2,3-didehydro-2-deoxy-N-acetylneuraminic acid, D9050, Sigma-Aldrich) was dissolved in H_2_O at a final concentration of 50 mM. These inhibitors were used at a concentration of 100 μM. The autophagic inhibitor ammonium chloride (NH_4_Cl, A3610500, AppliChem, Darmstadt, Germany) was dissolved in Phosphate-buffered Saline (PBS) at a final concentration of 200 mM and was used at a concentration of 10 mM.

### 4.2. Cytofluorimetric Analysis

Lectin binding assay was performed by biotinylated Sambucus Nigra (SNA) lectin and biotinylated Maackia Amurensis (MAL II)/FITC-Streptavidine indirect staining on U87-MG and U251 cells. Results were compared to extracellular PSA expression by PSA-NCAM/AlexaFluor 488 indirect staining. In brief, U87-MG cells (2 × 10^5^) and U251 cells (3 × 10^5^) were seeded into 60 mm diameter plates and maintained overnight. Then, the medium was removed, cells were washed twice in PBS, and the cells were grown in DMEM 0.2% FBS for 72 h. Afterwards, cells were collected, washed, and resuspended in Carbo Free Blocking solution (SP-5040, Vector Laboratories, Burlingame, CA, USA). Samples were incubated for 30 min at 4 °C with 10 µL of rabbit monoclonal anti-PSA-NCAM antibody (MBS488177, MyBioSource, San Diego, CA, USA—dilution 1:10), 1 µL of biotinylated Sambucus Nigra Lectin (SNA, B-1305, Vector Laboratories—dilution 1:100), or 1 µL of biotinylated Maackia Amurensis Lectin II (MALII, B-1265, Vector Laboratories—dilution 1:100) before being washed and resuspended again in 100 µL of PBS and 2% FBS. The samples stained for PSA were incubated for an additional 30 min at 4 °C with AlexaFluor 488-conjugated goat anti-rabbit antibody (dilution 1:200, A11034, Life Technologies, Carlsbad, CA, USA). The samples stained for SNA and MAL II were incubated for an additional 30 min at 4 °C with FITC-Streptavidin (dilution 1:500, 405201, Biolegend, San Diego, CA, USA).

To test the effect of autophagy inhibition on the extracellular expression of the NCAM and PSA, cytofluorimetric analysis was performed on U87-MG and U251 cells exposed to 0.2% FBS for 72 h in the presence or not of 10 mM NH_4_Cl following the above-mentioned protocol. After cell collection, samples were incubated for 30 min at 4 °C with 10 µL of rabbit monoclonal anti-PSA-NCAM antibody (MBS488177, MyBioSource—dilution 1:10) or 10 µL of APC-eFluor 780-conjugated anti-human CD56 (NCAM, 47-0567-41, eBioscience, Waltham, MA, USA) before being washed and resuspended again in 100 µL of PBS and 2% FBS. The samples stained for PSA were incubated for an additional 30 min at 4 °C with AlexaFluor 488-conjugated goat anti-rabbit antibody (dilution 1:200, A11034, Life Technologies).

In both cases, after being additionally washed with ice cold PBS, cells were resuspended in PBS and 2% FBS, and the samples were acquired on a Cytoflex SRT instrument using CytExpert SRT software (v.1.2, both by Beckman Coulter, Milan, Italy). At least 20,000 events were recorded and analyzed using FlowJo software (v.10.10.0, Becton Dickinson, Milan, Italy). Each experiment was performed independently three times.

### 4.3. Western Blot Analysis

Western blot analysis of U87-MG, U251, and primary cells’ total protein extracts was performed as previously reported with some modifications [73]. Briefly, cell pellets were lysed in a RIPA buffer (50 mM Tris–HCl pH 8.0, 150 mM NaCl, 1% Nonidet P-40, 1 mM EDTA, 0.5% sodium deoxycholate, 0.1% SDS) with protease inhibitors, 1 mM PMSF, 1 mM DTT, and 0.5 mM sodium orthovanadate (Sigma–Aldrich). Protein concentration was determined by the Bradford assay (Bio-Rad, Hercules, CA, USA). Then, 20 to 40 µg of total proteins per sample were resolved on SDS–PAGE gels and blotted onto a PVDF membrane (Amersham HyBond-P GE Healthcare, Chicago, IL, USA). After blocking at room temperature in 5% dry milk in Phosphate Buffer Saline (PBS, Sigma-Aldrich) containing 0.1% Tween-20 (Sigma-Aldrich) for 1 h, membranes were incubated overnight at 4 °C with the following primary antibodies: rabbit monoclonal anti-PSA-NCAM (MBS488177, MyBioSource—dilution 1:1000), rabbit monoclonal anti-LC3A/B (12741, Cell Signaling Technology, Danvers, MA, USA—dilution 1:1000), and rabbit polyclonal anti-p62 (5114, Cell Signaling Technology—dilution 1:1000); mouse monoclonal anti-α-tubulin (T5168, Sigma-Aldrich—dilution 1:5000) antibody was used to normalize results. Membranes were then incubated with anti-mouse and anti-rabbit horseradish peroxidase (HRP)-conjugated secondary antibodies (170-6516, 170-6515, Bio-Rad—dilution 1:15,000). Signals were detected by Clarity ECL Western Blotting substrates (170-5060, Bio-Rad). Digital images were acquired using a ChemiDoc XRS C System (BioRad). Band intensities were quantified by densitometric analysis using Image Lab software (v.6.1, BioRad), and the relative adjusted volumes were normalized to those of α-tubulin. Each experiment was performed independently three times and results are expressed as mean ± SD.

### 4.4. RNA Extraction and Real-Time PCR

Total RNA was isolated from U87-MG and U251 cells using the Total RNA Purification Kit (Norgen Biotek Corp., Thorold, ON, Canada) according to the manufacturer’s instructions. To extract RNA, GBM cell lines were seeded into 60 mm diameter plates and maintained overnight. Subsequently, the medium was removed, cells were washed twice in PBS and grown in DMEM 0.2% FBS for 72 h. Then, mRNA concentration was quantified using a Nanodrop spectrophotometer (ThermoFisher Scientific, Waltham, MA, USA). One microgram of mRNA was converted to cDNA using the High-Capacity cDNA Reverse Transcription Kit (Applied Biosystem, Warrington, UK) according to the manufacturer’s instructions. Gene expression was quantified by real-time PCR using the ViiA 7 real-time PCR System and Power SYBR Green PCR Master Mix (Applied Biosystem) according to the manufacturer’s instructions. Each experiment was independently repeated three times in triplicate and results are expressed as mean ± SD. Gene expression levels were calculated from real-time PCR data by the comparative threshold cycle (CT) method considering the HPRT1 housekeeping gene as an internal reference as already reported by our group [74]. The following gene-specific primers were used in the present study: human ST8SiaII (STX): FW 5′-CCTCATCTTCGCAGACATCTCA-3′, RV 5′-ATCTGATTGTACCTCTGCCTCC-3′; human ST8SiaIV (PST): FW 5′-ACTGAAAGTGCGAACTGCCT-3′, RV 5′-GAGAAGACCTGTGCTGGGTC-3′; human ST3Gal1: FW 5′-TTCCGGGAGCTGGGAGATAA-3′, RV 5′-GATCTTTGCAGGAACCGGG-3′; human ST3Gal6: FW 5′-GATTGTGGCTTGATGTGGCA-3′, RV 5′-GGGTTGTCAGGAGAGAGCTT-3′; human ST6Gal1: FW 5′-CCCCAGAAGAGATTCAGCCA-3′, RV 5′-TCTTCTCATAGAGCAGCGGG-3′; human ST6GalNAC5: FW 5′-GGATCCCAATCACCCTTAG-3′, RV 5′-TAGCAAGTGATTCTGGTTTCCA-3′; human CMAS: FW 5′-ACCTGGCAGCCCTAATTCTG-3′, RV 5′-TCGAAACCCATACACTCTGGAA-3′; human NEU1: FW 5′-ACCCCAATCAGCCCTTTTACA-3′, RV 5′-CTGGTCACACAGCGTCATCA-3′; human NEU2: FW 5′-CGGGCTTGATTTCCAGGAGT-3′, RV 5′-GTGTGGGGTGAGTGTAGAGC-3′; human NEU3: FW 5′-AATGTGAAGTGGCAGAGGTGA-3′, RV 5′-TCACAGAGCTGTCGACTCAGG-3′; human NEU4: FW 5′-TGCTGGTACCCGCCTACAC-3′, RV 5′-CCGTGGTCATCGCTGTAGAA-3′; human HPRT1: FW 5′-TGATAGATCCATTCCTATGACTGTAGA-3′, RV 5′-CAAGACATTCTTTCCAGTTAAAGTTG-3′.

### 4.5. Histological and Immunohistochemical Analyses

Immunohistochemical analysis was conducted as previously reported by our group [75], with some modifications. Formalin-fixed, paraffin-embedded tissue samples used for immunohistochemical analysis in this study were previously collected and characterized as part of earlier studies [30,72]. Briefly, paraffin-embedded tissues of patients diagnosed with GBM IDH-wild type (CNS WHO grade 4) and of a control donor were deparaffinized in xylene, rehydrated in descending graded alcohols, incubated for 15 min in 3% H_2_O_2_ in methanol to block endogenous peroxidases activity, and then subjected for 30 min to microwave heat-induced antigen retrieval in a sodium citrate buffer (10 mM tri-sodium citrate dihydrate, 0.05% Tween 20, pH 6.0). After a blocking step with the Super Block reagent (ScyTek Laboratories, Logan, UT, USA) for 10 min, different serial sections were incubated overnight with rabbit monoclonal anti-PSA-NCAM (MBS488177, MyBioSource—dilution 1:100) or rabbit monoclonal anti-LC3A/B (12741, Cell Signaling Technology—dilution 1:200) at 4 °C, washed three times with PBS, incubated for 10 min with UltraTek Anti-Polyvalent (ScyTek Laboratories) at room temperature, washed again three times with PBS, and incubated 10 min at room temperature with UltraTek HRP (ScyTek Laboratories). Then, slides were washed three times in PBS prior to being stained with 3-3-diaminobenzidine chromogen (DAB, ScyTek Laboratories) to visualize the reaction product. Finally, slides were counterstained with hematoxylin to visualize nuclei. Hematoxylin and eosin (H&E) staining was performed to evaluate the typical morphological alterations of GBM. A Nikon Eclipse Ni motorized microscope system at 20× magnification was used to acquire images. This study was carried out according to the principles of the Helsinki Declaration and the protocols approved by the ethics committee.

### 4.6. Statistical Analysis

All statistical analyses were performed using GraphPad Prism v.7 software. Results are expressed as a percentage of the mean ± standard deviation (SD). In all cases, data were analyzed by 1-way analysis of variance (ANOVA). A *p*-value <0.05 was considered as statistically significant.

## Data Availability

Main data generated or analyzed in this study are included in this article. Details are available from the corresponding author on reasonable request.

## Supplementary Materials

The supporting information can be downloaded at: https://www.mdpi.com/article/10.3390/ijms26157625/s1.
