# Supplementary figures and images for: Polysialylation of Glioblastoma Cells Is Regulated by Autophagy Under Nutrient Deprivation
Source: Int J Mol Sci. 2025 Aug 6;26(15):7625. doi: 10.3390/ijms26157625 (PMC12347492; doi:10.3390/ijms26157625)

U87MG

Ctrl

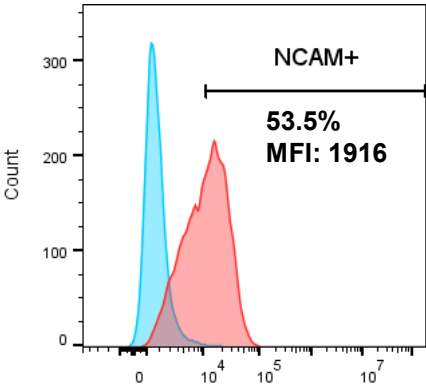

0.2% FBS

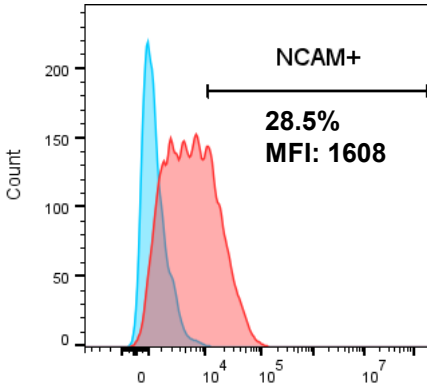

0.2% FBS  
NH<sub>4</sub>Cl 10mM

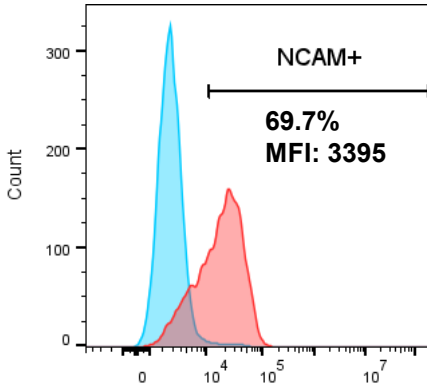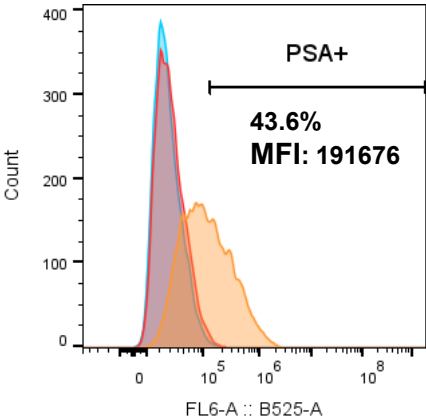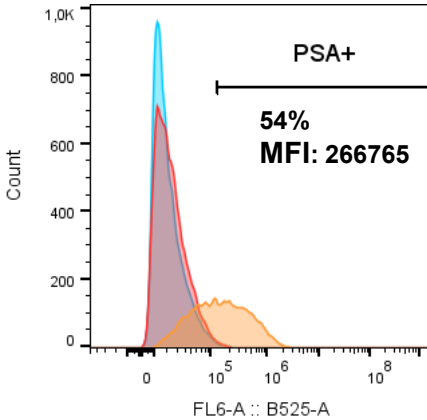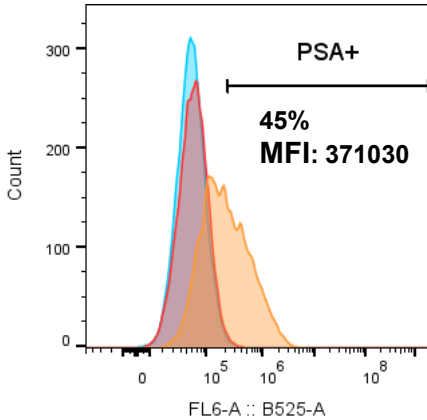

U251

Ctrl

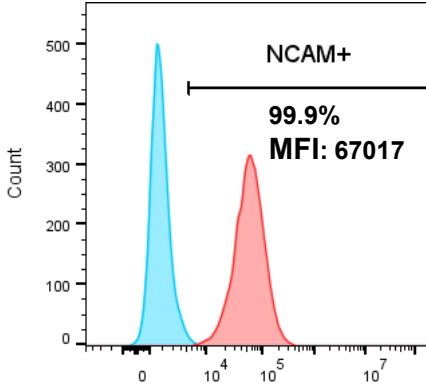

0.2% FBS

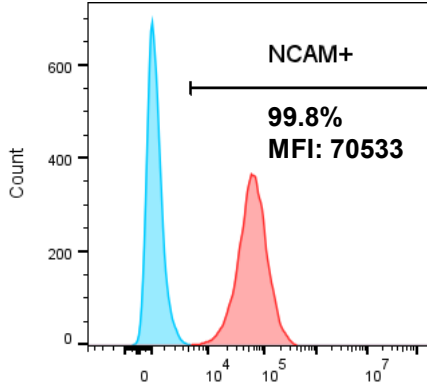

0.2% FBS  
NH<sub>4</sub>Cl 10mM

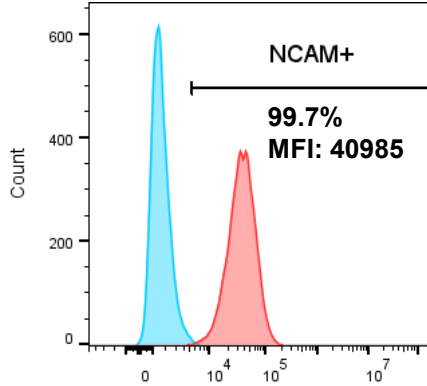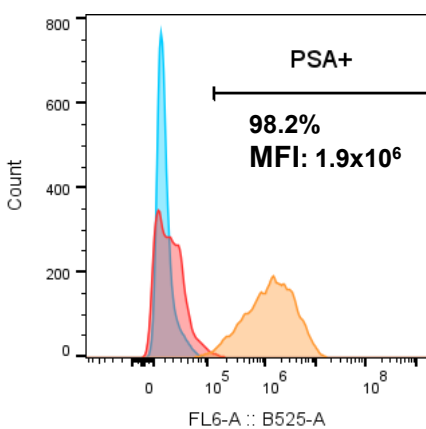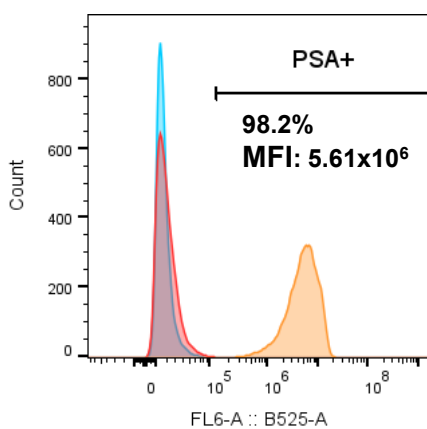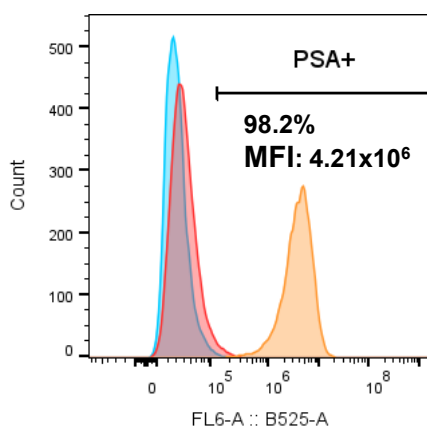

Supplement: Supplementary file 1 [file ijms-26-07625-s001.zip › Figure S1.pdf]
